# Release of Matrix-Bound Growth Factors; the role of increase in Sodium Concentration with Cartilage Compression

**DOI:** 10.1093/function/zqac028

**Published:** 2022-05-14

**Authors:** Jill Urban

**Affiliations:** Department of Physiology, Anatomy and Genetics, University of Oxford, OX1 3PT, Oxford, UK

**A Perspective on “Matrix-Bound Growth Factors are Released upon Cartilage Compression by an Aggrecan-Dependent Sodium Flux that is Lost in Osteoarthritis”**

Load-bearing cartilages do not appear to be very active tissues. They consist mainly of a dense porous matrix whose major constituents are water, fibrillar collagens and a meshwork of proteoglycans; they contain very few cells (chondrocytes). Yet throughout life, the chondrocytes integrate complex and varying extracellular signals to produce and maintain a matrix able to promote joint mobility yet support high and varying mechanical loads.^1^

Central to the mechanical behaviour of cartilages is role of the polyanionic proteoglycan aggrecan; its concentration of fixed negative charges (FCD) dictates the concentration of mobile ions in the matrix; anions are partially excluded and in normal adult articular cartilage, Na^+^ concentrations can be more than twice those in physiological fluids; a high Donnan osmotic pressure results.^2^ Fluid thus tends to be imbibed by cartilage to maintain osmotic equilibrium, but, in healthy adult human articular cartilage, tension developed by its almost inextensible collagen network resists swelling. The resultant swelling pressure, together with the low hydraulic permeability imparted by the dense network of proteoglycans, limits loss of hydration under load. By contrast, excised osteoarthritic cartilage, despite having a low intrinsic FCD because of aggrecan degradation, swells substantially; here the weakened collagen network is no longer able to restrain even the low swelling pressure developed by the degraded tissue.^3^

Articular cartilage routinely experiences high and varying mechanical loads. Chondrocytes respond to the resulting mechanical signals to produce a cartilage matrix able to withstand customary loading patterns; injurious loading levels can, however, induce matrix degradation and lead to osteoarthritic changes. The cartilage matrix of loaded areas has a higher aggrecan content than relatively unloaded areas of the same joint. Moreover, when loading is removed, chondrocytes respond by reducing rates of aggrecan synthesis and increasing production of aggrecan-degrading proteases; aggrecan concentrations can fall substantially.^4^

How chondrocytes sense load and regulate turnover of the cartilage matrix has thus been of longstanding interest. An understanding of mechanotransduction mechanisms is advancing rapidly and is summarised in recent reviews.^1^ Briefly, chondrocytes sense the load-induced changes to the matrix through multiple mechanosensitive integrins, membrane ion transporters and channels which mediate pathways of biosynthesis. The influences of loading on aggrecan production have been best characterized, but physiological loading regimes have also been found to change synthesis rates of a number of other matrix macromolecules and growth factors, while production of agents involved in matrix degradation tend to be upregulated under abnormal mechanical environments. In general synthesis of matrix molecules is stimulated under high frequency dynamic loading but is depressed under regimes where fluid is expressed. Indeed chondrocytes appear very sensitive to even slight changes in customary hydration levels and the consequent changes in FCD.^5^ Changes from in-situ extracellular osmotic and ionic environments alter cell volumes, depress rates of matrix production, and induce production of inflammatory factors in a dose-dependent manner, while osmotic shock on cutting injury can lead to chondrocyte death and degradation of adjacent matrix; this response is mitigated by bathing the cut in hyper-osmotic sucrose-NaCl solutions.

The recent study of Keppie et al.^6^ has uncovered another role of the FCD in mediating the chondrocyte's responses to mechanical load. Here, rather than signalling directly to the chondrocyte, a hyper-physiological compressive loading regime which expressed water, thus increasing extracellular Na^+^ levels as confirmed by microscale ^23^Na MRI, promoted bioavailability of growth factors; these growth factors are normally inactive and sequestered by heparan sulphates in the pericellular matrix. Growth factors were also released from normal cartilage, whether live or dead, after cutting injury, with extent of release increasing with increase in NaCl concentration of the incubating medium. However, in severely osteoarthritic or IL-1 treated cartilages which, because of aggrecan loss, have low interstitial Na^+^ concentrations, release of growth factors was only seen when NaCl concentrations were high. These results all suggest growth factor release can be promoted by a Na^+^ dependent mechanism which can be mechanically induced.

Several mechanisms, among them mechanical stress, have been proposed for the release of the substantial reservoir of growth factors sequestered in the extracellular matrix.^7^ The role of mechanical stress in mediating growth factor bioavailability through modulating interstitial Na^+^, provides a new approach. However, is the increase in Na^+^ concentration under load alone sufficient to cause growth factor release? Is release specific to Na^+^; concentrations of other cations, (e.g., Ca^++^, H^+^), will also increase with fluid expression. Moreover, release was only seen in areas where either severe compression or cutting injury disturbed the collagen network and altered collagen tension,^6^ whereas in intact, unloaded devitalized cartilage plugs, others have reported that even high exogenous NaCl concentrations did not promote TGF-β release.^8^ Tensile-dependent release mechanisms for latent TGF-β have been identified in other tissues;^7^ could such mechanisms contribute to Na^+^ mediated release of the growth factors studied by Keppie et al.? Indeed, could changes to the tensile properties of the collagen network, found even in early osteoarthritic cartilage,^9^ also contribute to the failure to release growth factors from the highly degenerate aggrecan-depleted osteoarthritic cartilage specimens? Although in animal experiments, proteoglycans were substantially (20%–60%) depleted from unloaded cartilages, these cartilages, unlike osteoarthritic cartilages, were able to repair once loading was restored;^4^ in these cartilages, unlike in IL-1 treated or osteoarthritic cartilages, the collagen network was virtually unaffected.

The work of Keppie et al.,^6^ has so far only found loading-induced bioavailability, identified by SMAD2 expression, in the severely compressed surface zone where Na^+^ concentrations were much higher than in the deeper cartilage zones, suggesting growth factor release depended on Na^+^ concentrations increasing above a critical threshold value. However, TGF-β release and SMAD2 expression have also been reported in cartilage plugs exposed to physiological levels of dynamic loading^10^ where any increase in Na^+^ concentrations is likely to be small. Determination of how loading regimes regulate Na^+^ concentrations and thus mediate growth factor release and activation could help understand whether this newly uncovered mechanism is only important under conditions of cartilage injury, or if it also promotes growth factor release during routine physiological loading and hence is involved in load-induced maintenance of normal cartilage composition.

## Data Availability Statement

No new data were generated or analyzed in support of this research.
